# An immunogenic cell death-related classification predicts prognosis and response to immunotherapy in kidney renal clear cell carcinoma

**DOI:** 10.3389/fonc.2023.1147805

**Published:** 2023-08-23

**Authors:** Licheng Wang, Yaru Zhu, Zhen Ren, Wenhuizi Sun, Zhijing Wang, Tong Zi, Haopeng Li, Yan Zhao, Xin Qin, Dacheng Gao, Libo Zhang, Ziyang He, Wei Le, Qiang Wu, Gang Wu

**Affiliations:** ^1^ Department of Urology, Tongji Hospital, School of Medicine, Tongji University, Shanghai, China; ^2^ School of Biomedical Engineering, Dalian University of Technology, Dalian, China; ^3^ Research Center for Translational Medicine, Shanghai East Hospital, Tongji University School of Medicine, Shanghai, China; ^4^ Department of Obstetrics and Gynecology, Tongji Hospital, School of Medicine, Tongji University, Shanghai, China; ^5^ Department of Gastroenterology and Hepatology, Tongji Hospital, School of Medicine, Tongji University, Shanghai, China

**Keywords:** immunogenic cell death, kidney clear cell carcinoma, immune, prognostic assessment, tumor mutation burden

## Abstract

**Introduction:**

Immunogenic cell death (ICD) is a form of regulated cell death that activates an adaptive immune response in an immunocompetent host and is particularly sensitive to antigens from tumor cells. Kidney clear cell carcinoma (KIRC) is an immunogenic tumor with extensive tumor heterogeneity. However, no reliable predictive biomarkers have been identified to reflect the immune microenvironment and therapeutic response of KIRC.

**Methods:**

Therefore, we used the CIBERSORT and ESTIMATE algorithms to define three ICD clusters based on the expression of ICD-related genes in 661 KIRC patients. Subsequently, we identified three different ICD gene clusters based on the overlap of differentially expressed genes (DEGs) within the ICD clusters. In addition, principal component analysis (PCA) was performed to calculate the ICD scores.

**Results:**

The results showed that patients with reduced ICD scores had a poorer prognosis and reduced transcript levels of immune checkpoint genes regulated with T cell differentiation. Furthermore, the ICD score was negatively correlated with the tumor mutation burden (TMB) value of KICD. patients with higher ICD scores showed clinical benefits and advantages of immunotherapy, indicating that the ICD score is an accurate and valid predictor to assess the effect of immunotherapy.

**Discussion:**

Overall, our study presents a comprehensive KICD immune-related ICD landscape that can provide guidance for current immunotherapy and predict patient prognosis to help physicians make judgments about the patient’s disease and treatment modalities, and can guide current research on immunotherapy strategies for KICD.

## 1 Introduction

ICD is a form of regulated cell death that activates an adaptive immune response in immunocompetent hosts (1, 2). ICD can be caused by a variety of stimuli, including viral infections, chemotherapeutic agents, and radiation therapy, and is particularly sensitive to antigens derived from tumor cells (3). The concept of cancer immunotherapy is to use the immune system to trigger an anti-tumor immune response (4). As a result, ICD is now being used in several preclinical models for anticancer chemotherapy, and clinical evidence suggests that tumor-specific immune responses can help improve the efficacy of conventional chemotherapeutic agents (5, 6).

The incidence of kidney cancer is on the rise globally, especially in the younger population (7, 8). In 2020, there were more than 431,000 new cases of kidney cancer and 179,000 deaths worldwide (9). Kidney clear cell carcinoma (KIRC) is the most common histologic cluster, with extensive tumor heterogeneity (10). KIRC is frequently genetically altered, such as somatic mutations in VHL, PBRM1, SETD2, BAP1, KDM5C, and PI3K-AKT-mTOR pathway genes (11). Although nephrectomy has shown good efficacy in the treatment of localized KIRC, more than 30% of patients experience advanced disease progression and 25% eventually experience disease recurrence (12). As KIRC is considered an immunogenic tumor, many different immunotherapeutic approaches have been tried in the past (13). Despite being strongly infiltrated, immune dysfunction promotes renal tumor growth and evasion. The tumor-induced changes in Dendritic Cells (DC) cell differentiation and the induction of anergy-associated genes in T cells can partially explain the impaired antitumor response (14). In recent years, treatment options for advanced KIRC have changed dramatically with the advent of targeted agents and immune checkpoint inhibitors (PD-1). However, the fact remains that real-life clinical practice still faces the enormous challenge of optimizing individualized treatment strategies. It is well known that biomarkers and predictive models can be used to predict risk stratification and case selection for targeted therapies, immunotherapies and combination therapies (10). However, to date, no reliable predictive biomarkers have been identified to reflect the immune microenvironment and treatment response in KIRC (15). Therefore, a more detailed grouping of KIRCs has important implications for guiding treatment (16).

Therefore, in this study, we first explored the correlation between KIRC and ICD using relevant tools. Then, two computational algorithms, CIBERSORT and ESTIMATE, were used to analyze the expression of ICD-related genes in tumor samples. In addition, we classified KIRC into three clusters based on the differences in the expression of ICD-related genes. Finally, in this study, we identify ICD-related biomarkers in KIRC and develop an ICD scoring mechanism that allows for an overall evaluation of the immune microenvironment and prognosis of KIRC patients, as well as for assessing their response to immunotherapy. In the future, this technology could help physicians make important judgments about patient condition and treatment modalities.

## 2 Materials and methods

### 2.1 Data collection and pre-processing

A total of 621 transcriptome data samples of KIRC samples were collected from The Cancer Genome Atlas (TCGA) database and International Cancer Genome Consortium (ICGC) database. Transcripts Per Kilobase per Million mapped reads (TPM) of TCGA-KIRC and ICGC-KIRC was derived from the University of California Santa Cruz (UCSC) Xena browser (https://xenabrowser.net/datapages/). The dataset used R “Combat” algorithm to eliminate the batch effect from the non-biological technical biases of each dataset (16). In addition, samples of clinical information such as age, gender, tumor stage, and survival time were collected, too. Further, the immune cell infiltration and somatic mutation data were also collected.

### 2.2 Unsupervised clustering analysis of KIRC

We were used to quantifying the infiltration levels for distinct immune cells in KIRC with the R CIBERSORT package (17). The ESTIMATE algorithm was utilized to calculate the expression of CRG for each KIRC sample to classify the CRG pattern and the immune score was also analyzed (18). We executed hierarchical agglomerative clustering of KIRC based on each CRD pattern. “Consensu Cluster Plus” R package was used to determine the number of clusters and stability, which is based on the unsupervised clustering “Pam” method according to Euclidean and Ward’s linkage and 500 times repeats to confirm the clustering stability.

### 2.3 Identification of DEGs related to the ICD phenotype

KIRC samples were classified into different ICD clusters based on the expression of ICD -related genes. R “limma” package was used to identify the differentially expressed genes (DEGs) among ICD clusters and the cutoff criteria were determined as P< 0.05 (adjusted) and |Log fold-change| > 1 (19). Further, the pathways of the DEGs enriched were constructed by DAVID and reflected by R “clusterProfiler” package. The Gene Ontology (GO) and Kyoto Encyclopedia of Genes and Genomes (KEGG) pathways were analyzed.

### 2.4 ICD score construction

On the basis of unsupervised clustering of DEGs, the ICD score was computed to quantify the ICD model for each KIRC individual. The KIRC patients were classified into distinct ICD gene clusters based on overlapped DEGs. R Boruta algorithm was further utilized for reducing the dimensionality of the different ICD gene signatures (20) and principal component 1 was extracted as the signature score by employing the principal component analysis (PCA). Finally, we defined the ICD score for each patient by employing the gene expression grade index (21).


ICD score=∑KIRC1A−∑KIRC1B

### 2.5 Somatic gene mutation in KIRC

The corresponding somatic mutation data of TCGA-KIRC and ICGC-KIRC was derived from the UCSC Xena browser, too. The tumor mutational burden (TMB) of each patient was counted by the total number of non-synonymous mutations in KIRC. To identify the correlation between somatic gene mutation and the ICD score, we grouped KIRC samples into low and high ICD score subgroups by “maftool” package of R software (22). Further, the top 20 driven mutation genes exploded.

### 2.6 Construction and validation of a predictive nomogram

For expanding the predictive ability of the ICD score, we built a nomogram according to the clinical information of TCGA-KIRC and ICGC-KIRC including, age, gender, and tumor stage. Further, a calibration method was used to verify the nomogram.

### 2.7 Statistical analysis

R version 4.0.5 was conducted for all statistical analyses. The two groups’ comparisons were carried out using the Wilcoxon test, and the Kruskal-Wallis test was employed for more than 3 groups. Kaplan-Meier (K-M) plot was conducted to generate survival differences with the log-rank test in patients with KIRC. Pearson correlation analysis was used to analyze the correlation coefficient. A P-value less than 0.05 indicates statistical significance. The analysis between ICD score subgroups and somatic mutation frequency was used a chi-square test analyzed the correlation, and the Pearson correlation analysis calculated the correlation coefficient. Two-tailed P< 0.05 was considered statistically significant.

### 2.8 Cell culture

KIRC cell line SW839 and renal epithelial cell line HEK-293T(RRID : CVCL_0063) were all obtained from American Type Culture Collection (ATCC, Rockville, MD, USA). Cells were cultured in RPMI-1640 containing 10% fetal bovine serum (FBS, Gibco, Carlsbad, CA, USA), 1% penicillin-streptomycin (HyClone, Logan, UT, USA), and then preserved in the atmosphere with 5% carbon dioxide (CO2) at 37°C.

### 2.9 Cell transfection

Plasmids were commercially purchased from Youze Biological Corporation. As per the manufacturer, cell transfection was performed using Lipofectamine 2000 (Invitrogen, Inc., CA, USA).

### 2.10 Quantitative real-time PCR analysis

The total RNA was extracted using TRIzol reagent. An equivalent of 1 µg of total RNA was subjected to reversed transcription into cDNA using PrimeScript™ RT reagent Kit (Takara, Kyoto, Japan) and Mir-X™ miRNA qRT-PCR SYBR® Kit (Takara, Kyoto, Japan). The mRNA expression of the target gene was determined by qRT-PCR conducted on the ABI-7900 system with SYBR Green (Takara, Kyoto, Japan). The expression of a target gene was normalized to that of GAPDH.

### 2.11 Transwell assay

A Transwell chamber (24-well) with Matrigel was applied to determine cell invasion following the manufacturer’s instructions. 200 µl transfected SW839 cells (1×105) were added in the upper chamber with RPMI1640 medium without FBS. 500 µl RPMI-1640 medium containing 10% FBS was added to the lower chamber. Cells were cultured for 24 h at 37 °C with 5% CO2. The cells were fixed with formaldehyde for 15 min and then stained with 0.1% crystal violet for 15 min. The invasive cell number was counted using a Zeiss Microscope (Nikon Corporation, Japan).

### 2.12 Cell counting kit

Cells were selected in the logarithmic growth phase and seeded in 96-well plates at 2 × 103 cells/well for 0h, 24h, 48h, and 72h. 10μL CCK8 solution (Dojindo, Japan) was added and the plate was incubated at 37°C for 2h in the dark. The absorbance was detected at 450 nm using a microplate reader (Thermo Fisher Scientific).

### 2.13 Wound healing assay

Cells were plated in 6-well plates and scratched vertical wounds with 10 μl tips after completely adherent. Cells were cultured in serum-free RPMI-1640 and photographed at 0h and 48h.

## 3 Results

### 3.1 ICD-related gene mutations associated with kidney cancer

**Figure 1:**
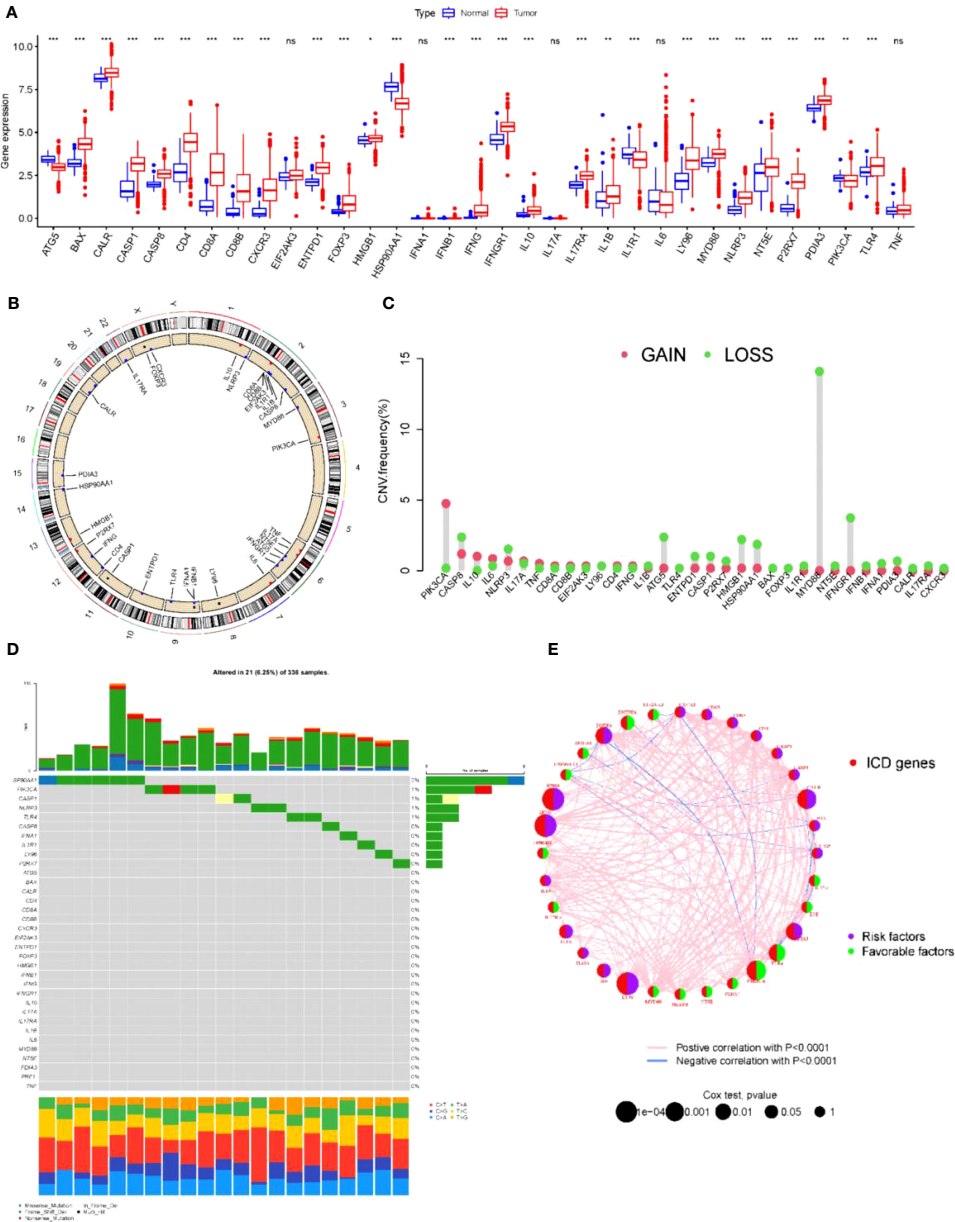
ICD-related gene mutations associated with kidney cancer **(A)** Expression of ICD relative genes in normal and KICD tumor tissues in patients *P< 0.05, **P< 0.01 *** *P*< 0.001. **(B)** Chromosomal localization of ICD-related genes in the overall population. **(C)** Gain and Loss of ICD-associated genes in the overall population. **(D)** Specific mutation patterns and base substitution patterns of ICD-related genes in human populations. **(E)** Single-factor cox analysis and co-expression analysis of ICD-related genes. ns, no significance.

First, the expression of ICD-related genes was compared in renal clear cell carcinoma tissues and normal tissues, and it was found that the expression of most ICD-related genes in renal clear cell carcinoma was significantly different from normal tissues (**Figure 1A**). Since the direct cause of the difference in expression is copy number variation and associated gene mutations, the copy number and mutations of ICD-related genes in the overall population were further investigated. ICD-related genes were localized in the chromosomes of the overall population (**Figure 1B**) and the associated copy mutation patterns were clarified (**Figure 1C**), where deletions of genes including MYD88, IFNGR1, and ATG5 were observed to be possibly associated with KIRC. Meanwhile, the specific mutation patterns and base substitution patterns of ICD-related genes in the population were specifically analyzed (**Figure 1D**), indicating that the high mutation rates of genes such as SP90AA1 and PIK3CA may be associated with KIRC. To further clarify the relationship between mutations in ICD-related genes and KIRC, one-way cox analysis and co-expression analysis of ICD-related genes showed that both the deletion and mutated genes just mentioned are oncogenes and have strong interactions with most other ICD-related genes associated with cancer (**Figure 1E**). Therefore, it is believed that there is a correlation between the degree of ICD-related gene expression and KIRC.

### 3.2 Differential ICD gene expression in KIRC

**Figure 2:**
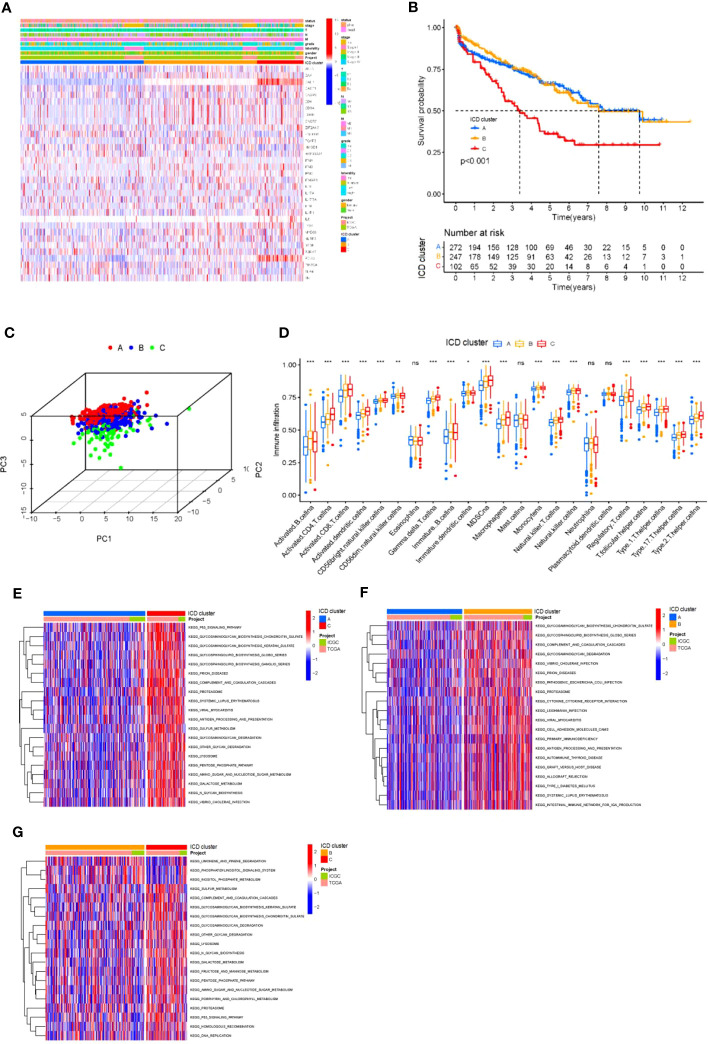
Differential ICD gene expression in KIRC **(A)** Unsupervised clustering of ICD-related genes in two independent KIRC cohorts. Rows represent ICD-related genes, and KIRC columns represent samples. **(B)** Kaplan-Meier curves for overall survival (OS) of all KIRC patients with ICD cluster classes. Log rank test showed an overall p< 0.001. **(C)** Three- dimensional diagram of principal component analysis (PCA) of ICD clusters. **(D)** The difference in the infiltration of 23 immune cells, immune score, and stromal score in three distinct ICD clusters. *P< 0.05, **P< 0.01 ***P< 0.001. **(E-G)** The pathways enriched by three ICD clusters using KEGG pathway enrichment analysis and identified by the “GSVA” R package. ns, no significance.

First, we used the ESTIMATE algorithm to quantify the expression levels of ICD-related genes in KIRC tumor tissues. Based on 621 tumor samples with ICD features (array expression database: TCGA & ICGC; The Cancer Genome Atlas TCGA-KIRC and ICGC-KIRC), unsupervised clustering using ConsesusClusterPlus with R software was used to classify KIDC patients into different clusters. We identified three independent ICD clusters with significant survival differences (log-rank test, p = 0.018; **Figures S1**, **2A**). To verify the validity of the grouping, the three clusters were tested separately for survival, and the survival of clusters A and B was significantly better than that of cluster C (**Figure 2B**). Then, the three clusters were subjected to 3D compositional clustering analysis, and it was observed that although the correlation of ICD-related genes was higher, the clustering analysis of different clusters indicated that the genes within the group were more closely related and correlated (**Figure 2C**), which also indicated the existence of some scientific validity and reasonableness of the grouping. Next, different clusters of tumors were counted for immune cell infiltration, and although the overall trends were similar across subgroups, there were significant within-group differences in the majority of types of immune infiltrating cells, including myeloid-derived suppressor cells (MDSCs), CD8+ T cells, and monocytes (**Figure 2D**), suggesting that the grouping by this D, suggesting that the classification of KIDC into 3 clusters by this grouping has its own biological significance. To further understand the biological significance corresponding to these three clusters and their differences, the three clusters were grouped in two and their related pathways were analyzed and compared (**Figures 2E–G**). The comparison revealed that the differences were more obvious in the comparison of clusters A and C; clusters A and B showed differences in pathway enrichment despite similar survival curve trends; while clusters B and C, in contrast to A and B, had large differences in survival analysis while similar or identical possibilities existed in the pathways. Therefore, although these three clusters may have their corresponding biological significance, there are still problems such as similar pathways and insignificant differences in survival curves. Therefore, we believe that these three clusters can be further analyzed for further treatment.

### 3.3 Reconstruction of tumor clusters

**Figure 3:**
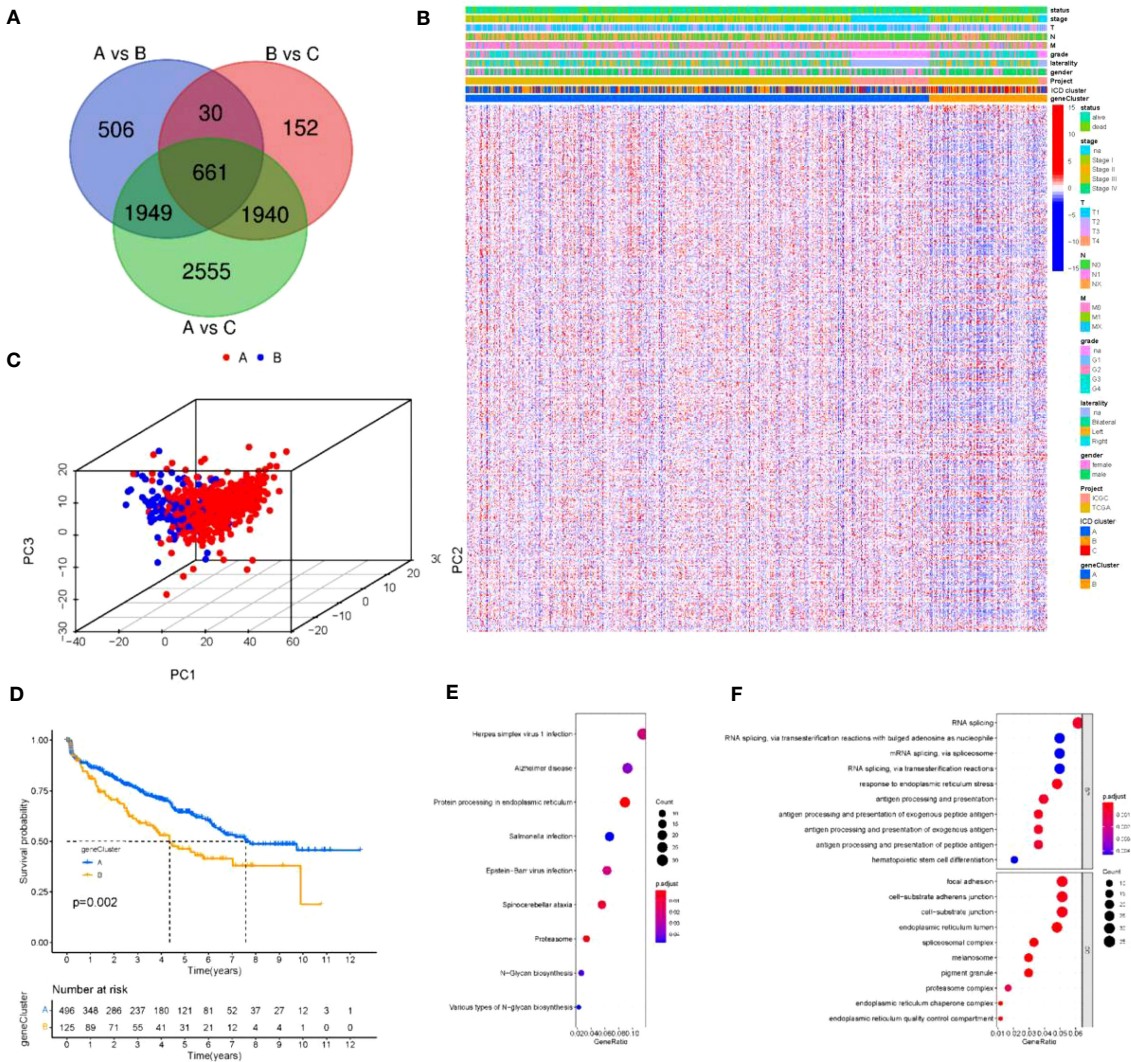
Reconstruction of tumor clusters **(A)** Venn diagram illustrating the number of DEGs among the three ICD clusters. **(B, C)** Gene Ontology (GO) and KEGG enrichment analysis of the ICD-relevant signature genes. The size of the dot represents gene count, and the color of the dot represents (p. adjust-value); **(D)** Unsupervised clustering of common DEGs among three ICD cluster groups to classify patients into three groups: gene clusters **(A–C, E)** Two- dimensional diagram of principal component analysis (PCA) of gene clusters. **(F)** Kaplan-Meier curves for overall survival (OS) of all KIRC patients with gene clusters. Log rank test showed an overall p =0.002.

To further optimize the typing of tumor clusters and reduce noise and redundancy, we re-screened all ICD-related genes and selected 661 genes that were significantly different in all three clusters A, B and C for more accurate analysis. We extracted 661 ICD-related genes and then performed GO and KEGG analysis(**Figure 3A**). GO analysis showed that the red module was highly enriched in the biosynthesis of multiple viral infections and proteins(**Figure 3B**). Additionally, signaling pathway analysis suggested that the ICD-related genes were enriched in exogenous antigen, peptide antigen, and other antigen processing and presentation, RNA shearing and splicing, cell-matrix attachment, and other physiological aspects related to tumor antigen immunity (**Figure 3C**). After confirming that these 661 ICDs were associated with tumor immunity and other physiological activities, we subjected these 661 genes to further analysis. The 661 ICD-related gene expressions were downscaled using the Boruta algorithm, and the samples were reclassified into two groups according to these 661 gene expressions, and the heat map was drawn in this way (**Figure 3D**). Then, the two gene clusters were clustered using 3D cluster analysis, and it was found that the two gene clusters differed in clustering (**Figure 3E**). In addition, to explore the prognostic significance of the clusters, we integrated the ICD gene clusters with survival information, and the survival of group A was significantly better than that of group B (**Figure 3F**).

### 3.4 Construction of the ICD score

**Figure 4:**
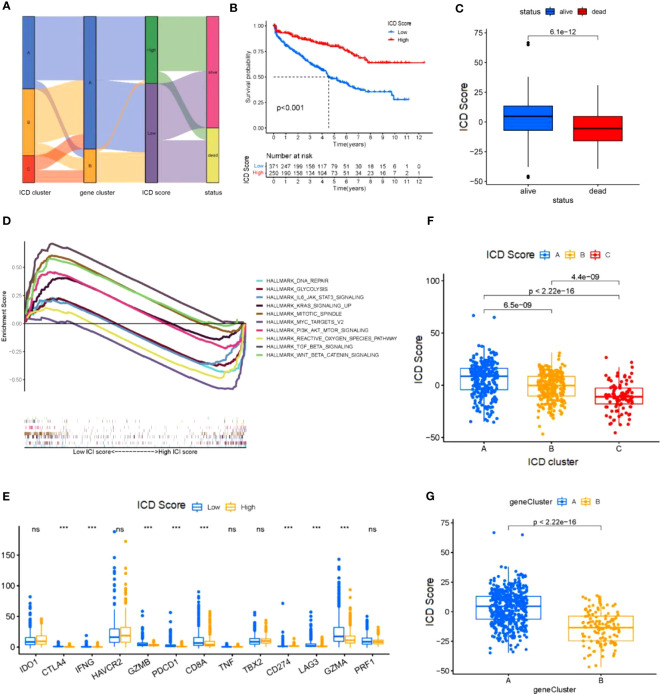
Construction of the ICD score **(A)** Sankey diagram shows the correlation between ICD cluster,gene cluster, ICD score, and status of KIRC patients. **(B)** Kaplan-Meier curve was used to predict the prognosis of the ICD score (log-rank test P< 0.001). **(C)** The regression of KICD patients with high ICD scores compared with KICD patients with low ICD scores (log-rank test **(B)** P=2.5*10^-13^). **(D)** KICD patients in the low and high ICD score subgroups. **(E)** Correlation of the ICD score with immune cellular. **(F)** The expression of immune-checkpoint genes and immune-activity genes in patients in the high and low ICD score subgroups. *P< 0.05, **P<0.01, ***P< 0.001. **(G)** ICD score distribution in gene cluster **(G)**. ns, no significance.

To obtain quantitative indicators of ICD status in KIRC patients, we used the principal-component analysis (PCA) to calculate specific scores for two groups: (1) ICD score A from ICD trait gene A, and (2) ICD score B from ICD trait gene B.We obtained the ICD score for each group by summing the score A and score B for each sample, and considered this score as the sum of individual scores. Finally, we defined the obtained score as the prognostic characteristics score of the ICD score. All patients were divided into two groups with high or low ICD scores by using the best cut-off values obtained with the “maftool” package of R software. The distribution of patients in the three gene clusters is shown in **Figure 4A**. To further ensure the reliability of the ICD scores, the two groups with high and low ICD scores were analyzed for survival (**Figure 4B**) and regression (**Figure 4C**), and it was clear that there was a significant survival difference(p<0.001). The gene set enrichment analysis (GSEA) test (**Figure 4D**) revealed that active pathways in the low ICD score group were associated with pro-tumor growth and positive regulation of mitosis, which positively explains the poor prognosis and regression of patients with low ICD scores. Also, in the high ICD score group, active reactive oxygen species metabolism and DNA repair pathways also represent better cellular activity and repair function, which implies a relatively high anti-tumor capacity of the organism. In addition, we generated correlation coefficient heat maps to visualize the prevailing landscape of immune cell interactions in ICD (**Figure 4E**). To further demonstrate the physiological significance of the scores, we performed expression analysis of 13 immune checkpoint-associated genes widely reported in the literature and found that although the expression trends of immune checkpoint-associated genes were the same across scores, there were still expression differences in eight genes, the vast majority of which were associated with regulation of T-cell differentiation (**Figure 4F**). Next, we substituted the scores into the three and two gene clusters in the previous section and observed statistically significant differences when the scores were substituted into the above gene clusters (**Figure 4G**), suggesting that the use of scores to represent patient conditions allows for a more detailed and specific evaluation of patient conditions than the use of genotyping alone.

### 3.5 Tumor mutational load correlates with ICD scores

**Figure 5:**
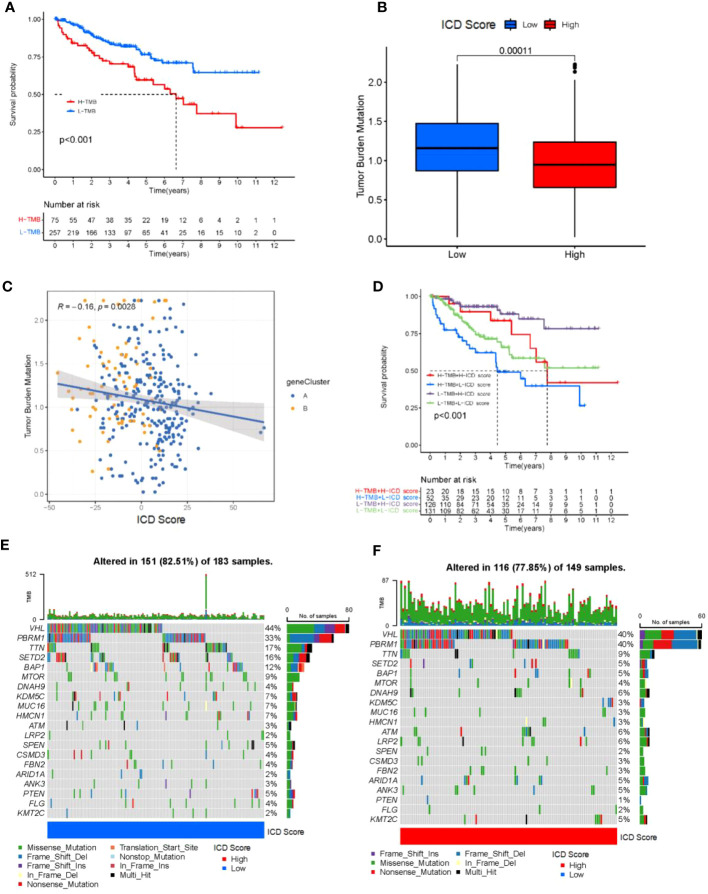
Tumor mutational load correlates with ICD scores. **(A)** Kaplan-Meier curve for low and high TMB subgroups in the ICD score cohort (log-rank test P<0.001). **(B)** Patients with high ICD score had high TMB value (P = 0.00011). **(C)** positive correlation was observed between ICD score and TMB (R = -0.16; P = 0.0028). **(D)** Kaplan-Meier curve for different subgroups (log-rank test P<0.001). **(E, F)** Oncoprint visualization of the top ten most frequently mutated genes in ICD-low cluster **(E)**, and ICD-high cluster **(F)**.

There is substantial evidence that CD8+ T cells infiltrating tumor tissue can recognize and eliminate tumor cells with high mutational load (nonsynonymous variants). This means that tumor-loading mutations (TMBs) may determine an individual’s response to cancer immunotherapy. Studies have shown that increased TMB is associated with improved response to PD-1 blockade and prolonged progression-free survival. Considering the clinical importance of TMB, to further understand it, we performed survival analysis and regression statistics for patients with different TMB (**Figures 5A, B**), and the results showed that TMB may be associated with patient prognosis. We sought to explore the intrinsic correlation between TMB and ICD scores by fitting the two and demonstrating a negative correlation (Spearman’s coefficient: R = -0.16, p = 0.0028; **Figure 5C**) Considering the prognostic value of the TMB and ICD scores, we next evaluated the synergistic effect of these scores in the prognostic stratification of KIRC. Stratified survival analysis showed that TMB status did not interfere with predictions based on ICD scores and that ICD score clusters showed significant survival differences in the high and low TMB subgroups (log-rank test, high TMB and high ICD scores (HH) versus high TMB and low ICD scores (HL), p< 0.001; **Figure 5D**) Through the analysis, we learned that the higher the tumor mutational load, the lower the ICD score, and the worse the prognosis of tumor patients, which is consistent with the previous description. Overall, these findings suggest that ICD scores may serve as a potential predictor independent of TMB and a valid measure of response to immunotherapy, and also suggest the value of ICD for further analysis.

In addition, we evaluated the distribution of somatic variants in KIRC driver genes between low and high ICD subgroups. The top 20 driver genes with the highest frequency of alteration were further analyzed, while their mutation patterns were shown (**Figure 5D**). Analysis of mutation annotation files from both databases revealed significant differences in the frequency of alterations between low and high ICD score groups for PBRM1, TTN, SETD2, BAP1, MTOR, KDM5C, MUC16, HMCN1, ATM, LRP2, SPEN, ARID1A, ANK3, PTEN, FLG and KMT2C (**Figure 5E**). Overall, patients with higher ICD scores had fewer overall tumor mutations, while the opposite was true for patients with lower ICD scores. These results may provide new ideas for studying the mechanisms of tumor ICD composition and gene mutations in immune checkpoint blockade therapy.

### 3.6 Significance of ICD score for patient prognosis and immunotherapy

**Figure 6:**
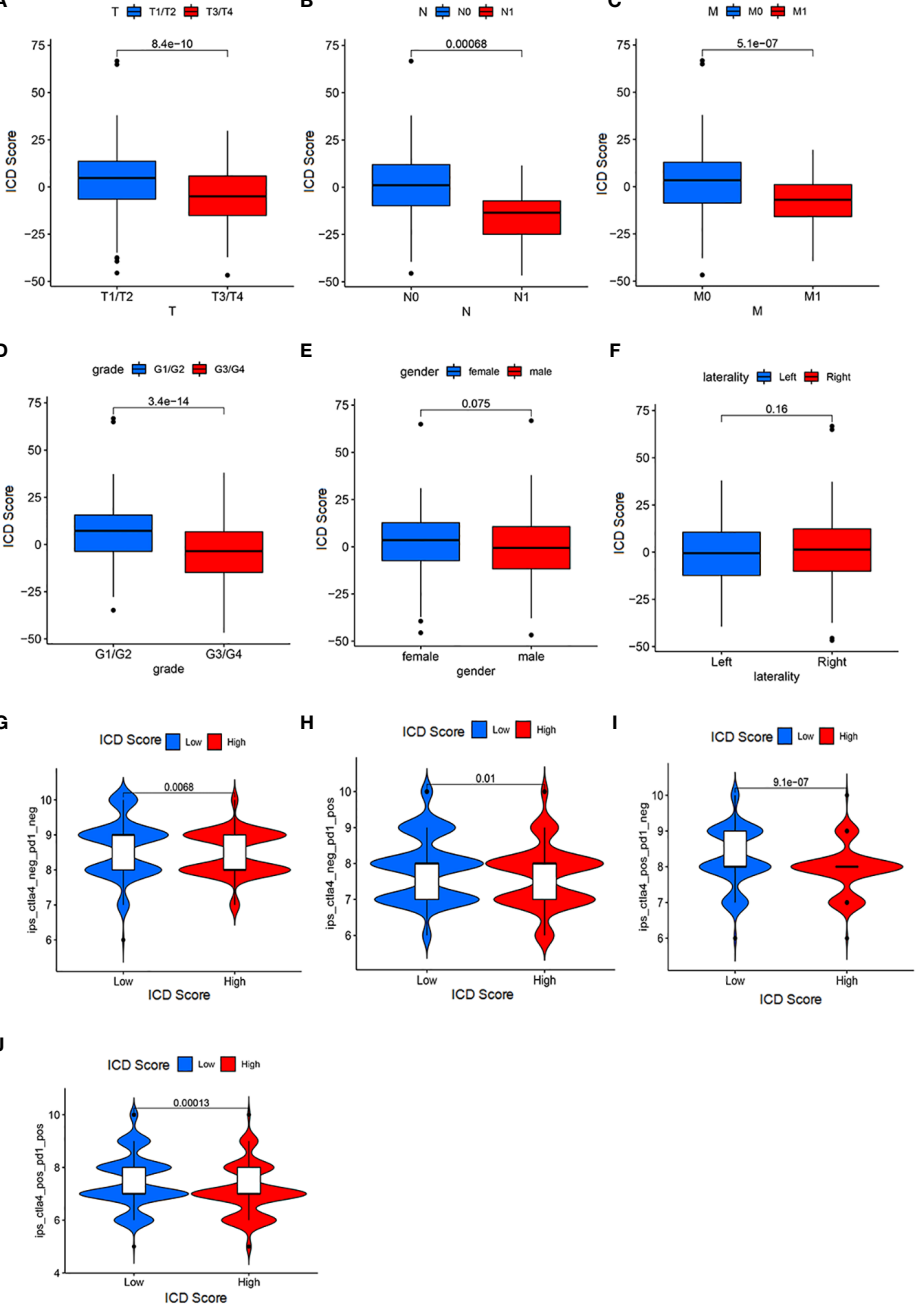
Significance of ICD score for patient prognosis and immunotherapy **(A)** The correlation between the T stage and ICD scores (Wilcoxon test, P = 3.7*10^-10^). **(B)** The association between the N stage and ICD scores (Wilcoxon test, P = 0.00061). **(C)** The association between the M stage and ICD scores (Wilcoxon test, P = 9.3*10^-8^). **(D)** The association between the grade and ICD scores (Wilcoxon test, P = 3.4*10^-14^). **(E)** The association between the M stage and ICD scores (Wilcoxon test, P = 0.033). **(F)** laterality parameters have no significant correlation with the ICI score. **(G)** Patients with a low ICI score have a better immune response to the IPS-CTLA4-neg-PD1-neg immunotherapy (log-rank test P = 0.0068). **(H)** Patients with a low ICI score have a better immune response to the IPS-CTLA4-neg-PD1-pos immunotherapy (log-rank test P = 0.001). **(I)** Patients with a low ICI score have a better immune response to the IPS-CTLA4-pos-PD1-neg immunotherapy (log-rank test P = 9.1*10^-7^). **(J)** Patients with a low ICI score have a better immune response to the IPS-CTLA4-pos-PD1-pos immunotherapy (log-rank test P = 0.00013).

The analysis above has clarified the existence of ICD scores for both their prognostic as well as immunotherapeutic implications. Therefore, to further relate to clinical practice, we used ICD score analysis including patients’ gender, age and their tumor staging, stage, metastasis and other clinical judgment indicators to understand the accuracy of ICD score in determining patients’ prognosis. By analysis we obtained that ICD scores were associated with age, gender, N fraction, and M fraction in patients with KIRC tumors (p<0.05; **Figures 6A–F**). In a subsequent analysis, we examined the utility of ICD scores in inferring patient treatment. For this purpose, the analysis was performed in the TCGA-ICGC cohort for patients receiving PD-L1 and CLTA4 immunotherapy in high versus low ICD scores. Notably, the objective remission rate (objective response) of anti-PD-L1 therapy was higher in the high ICD score group than in the low ICD group (p = 0.01; **Figure 6H**). And, the objective remission rate of anti-CLTA4 treatment was higher in the high ICD score group than in the low ICD group (p<0.005; **Figure 6I**). In addition, the same was observed in patients receiving both PD-L1 and CLTA4 immunotherapy (p<0.005; **Figure 6J**). Therefore, the ICD score can be used to determine the prognosis of immunotherapy in either patients receiving PD-L1 or CLTA4 immunotherapy alone or in patients receiving both PD-L1 and CLTA4 immunotherapy. Overall, these data suggest that ICD scores may correlate with response to immunotherapy. In addition, the ICD score remains a guide to prognosis for patients who do not receive both immunotherapies (p<0.005; **Figure 6G**).

**Figure 7:**
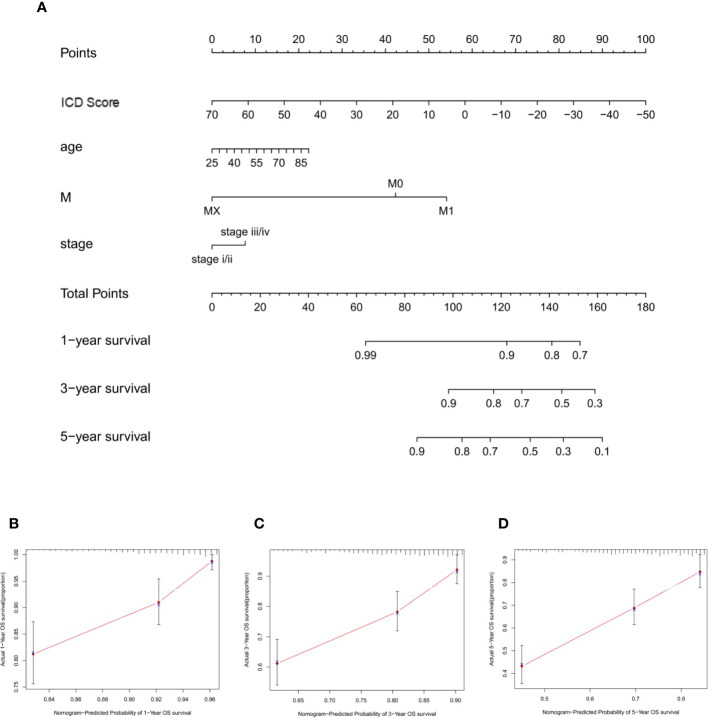
Construction of nomogram for ICD sroce. **(A)** The nomogram was based on ICD score, age, M stage, and T stage to predict the prognosis of 1, 3, and 5 years. **(B–D)** The 1-year **(B)**, 3-year **(C)**, and 5-year **(D)** calibration curves of the nomogram.

A nomogram based on clinical features was constructed using the “rms” R package to determine the prognostic value of the ICD score. Each parameter (ICD score, age, stage, and M stage) was assigned a point, and the total points were computed. Based on the total score, 1-, 3- and 5-year BCR- free survival rates were predicted (**Figure 7A**). The calibration plot validated that the nomograms couldpredict the prognosis of patients based on IC scores (**Figures 7B–D**).

### 3.7 RNF38 has an oncogenic effect in KIRC

**Figure 8:**
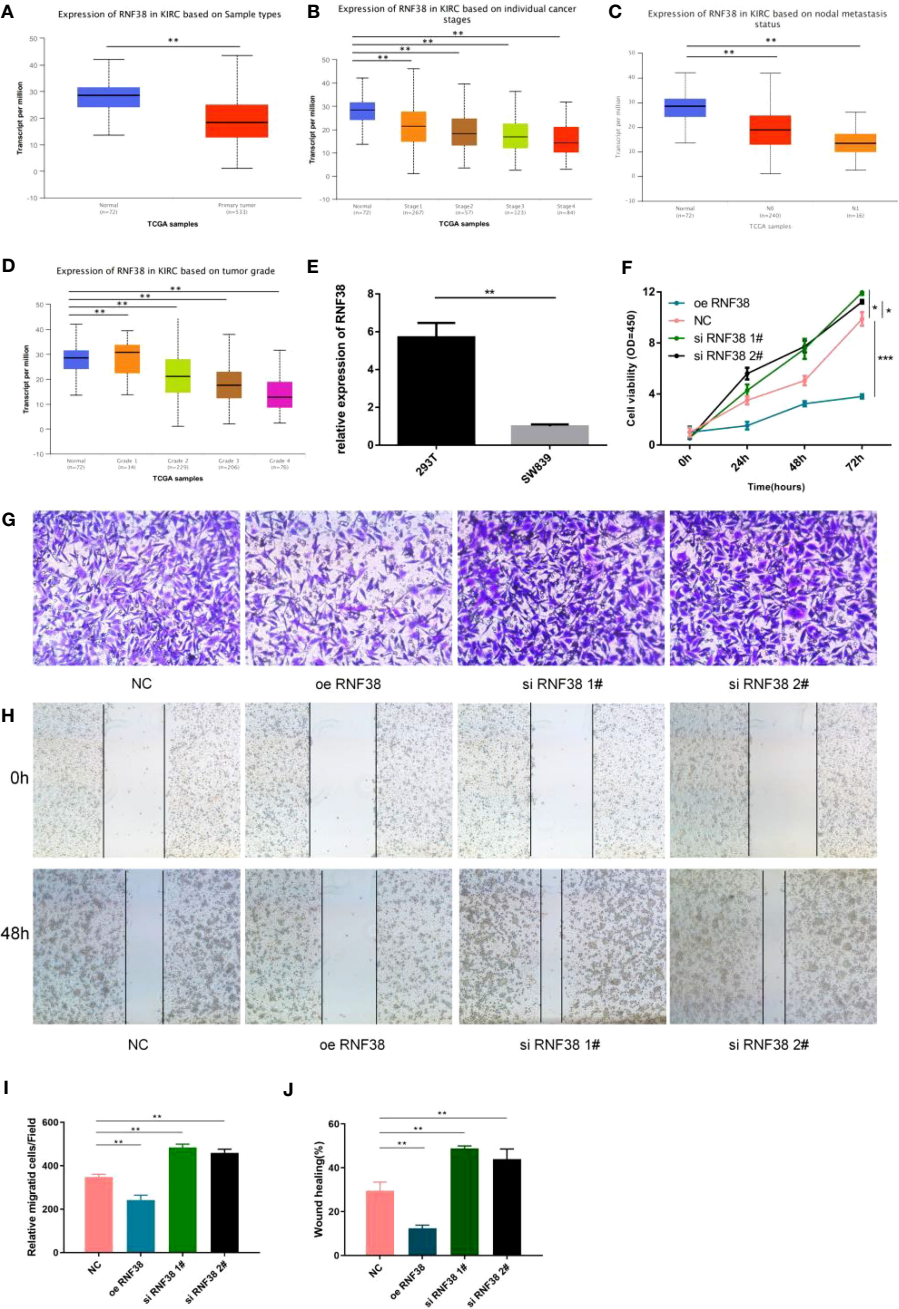
RNF38 has an oncogenic effect in KIRC **(A)** Expression of RNF38 in KIRC based on Sample types. **P<0.01. **(B)** Expression of RNF38 in KIRC based on individual cancer stages. **P< 0.01. **(C)** Expression of RNF38 in KIRC based on nodal metastasis status. **P< 0.01. **(D)** Expression of RNF38 in KIRC based on tumor grade. **P< 0.01. **(E)** Difference of RNF38 expression between 293T and SW839. **P< 0.01. **(F)** Comparison of the proliferation ability of normal SW839 cells, SW839 cells knocking down RNF38 and SW839 cells overexpressing RNF38. **(G, I)** Comparison of the invasive ability of normal SW839 cells, SW839 cells knocking down RNF38 and SW839 cells overexpressing RNF38.**P< 0.01. **(H, J)** Comparison of the migration ability of normal SW839 cells, SW839 cells knocking down RNF38 and SW839 cells overexpressing RNF38. **P< 0.01, ***p<0.001.

We selected RNF38, a gene with significant differences among the three groups, to validate the rationality of the scoring system. The expression level of RNF38 in tumor samples was significantly lower than that in normal tissue (**Figure 8A**). In addition, the expression of RNF38 decreased with the progression of the tumor stage (**Figures 8B–D**). By qPCR technique, we found lower expression of RNF38 in SW839 cells compared to HEK-293T cells (**Figure 8E**). We overexpressed RNF38 in SW839 cells and found that the proliferation (**Figure 8F**), invasion abilities (**Figures 8G, I**), and migration (**Figures 8H, J**) of the transfected cells were reduced. At the same time, knocking down RNF38 results in an increase in the above capabilities (**Figures 8F–J**). The above results corroborate the rationality of the grouping.

## 4 Discussion

Early clinical trials of immunotherapy have demonstrated its efficacy in treating low-risk and intermediate-risk KIRC and in extending progression-free survival in patients. However, an important limitation of immunotherapy is that only a small number of patients benefit from it. The KIRC guidelines for immunotherapy published by the Society for Immunotherapy of Cancer also emphasize that patients suitable for immunotherapy and their appropriate drug classes should be identified as early as possible (23). In this study, we established a method to quantify ICD associated gene mutations within tumors in KIRC. Our findings suggest that the ICD score is a valid prognostic biomarker and predictive indicator for assessing response to immunotherapy. There is growing evidence that immune prototype cell death within the KIRC affects immune cells and promotes tumor immunosuppression, leading to associated tumor survival and progression. In this study, we analyzed 621 KIRC samples for ICD-related genes, classified KIRC into three different clusters, and analyzed the scores found to correlate with patient prognosis. This emphasizes that preexisting immune responses have antitumor effects and positively influence the response to immunotherapy. Several seminal clinical and genomic studies have reported that KIRC is one of the tumor types that are highly infiltrated with immune cells. However, only some patients with KIRC respond to immunotherapy compared to patients with other tumor types with lower immune infiltration. This suggests that even the immune phenotype in the tumor does not absolutely predict response to immunotherapy. Thus, genetic analysis of KIRC has identified a series of mutations in ICD-related genes whose mutational status directly affects the expression of these genes, and changes in the expression of the genes involved may disrupt intercellular communication between infiltrating immune cells, thus shifting the balance between immune tolerance and sensitivity.

In the current study, we hypothesized that the combined characterization of ICD profiles and immune-related gene expression patterns would be a novel approach to develop patient-specific therapeutic strategies. We focused on ICD immune prototype-related genes of practical significance that can modulate the immune system, so we screened all ICD immune prototype-related genes into new ICD gene clusters to obtain immune-related genes. anti-tumor immune responses in ICD gene cluster A are associated with a good prognosis, and we hypothesized that patients in ICD gene cluster A might benefit from immunotherapy from immunotherapy. The results of our analysis are consistent with previous studies and suggest that the gene clusters in the current study may lead to the development of more precise immunotherapies.

Considering the individual heterogeneity of the immune environment, there is an urgent need to quantify the ICD patterns of individual tumors. Individual models based on tumor cluster-specific biomarkers have been well used in breast and colorectal cancers to improve prognosis (24–26). In the current study, with the help of Boruta algorithm, we built an individual-based model to improve outcome prediction. In the current study, with the help of the Boruta algorithm, we obtained potential “cluster biomarkers” and established an ICD score to quantify the ICD pattern. Through GSEA, we identified relevant physiological phenomena involved in immunosuppression, such as regulation of exogenous antigen, peptide antigen, and other antigen processing and presentation, RNA shearing and splicing, and cell-matrix attachment, and these genes were clearly enriched in the low ICD score group. Recently, preclinical reports have identified the relationship between gene mutations and response or tolerance the relationship between gene mutations and response or tolerance to immunotherapy (27, 28). The combined ICD scores at the genomic level showed significant differences in variant frequency between multiple genes with high and low ICD scores, and few of these genes were clearly associated with sensitivity or resistance.

By evaluating patients receiving immunotherapy, we found that ICD scores were significantly higher in patients who responded to immunotherapy, which validates its predictive value. Overall, this suggests that immunotherapy may be beneficial for patients with high ICD scores. Considering the activity of the TGF-b signaling pathway in the low ICD score cluster, TGF-b inhibition coupled with immune checkpoint blockade may be beneficial in patients with low ICD scores (29, 30). In addition, previous clinical studies have confirmed that synergistic treatment with TGF-b inhibitors and immune checkpoint inhibitors is more effective than single agent immunotherapy for solid tumors. In addition, there is an ongoing phase 1b/2 clinical trial (ClinicalTrials.gov: NCT02423343) testing the therapeutic efficacy of the combination of TGF-b and nivolumab in advanced solid tumors. Therefore, we consider the ICD score as a way to determine the prognosis of KIRC patients for immunotherapy. However, the results of the current study need to be validated in a larger cohort of KIRC receiving immunotherapy based on clinical trials.

In summary, we have comprehensively analyzed the ICD profile of KIRC to provide a clear picture of the regulation of anti/pro-tumor immune responses in KIRC. In response regulation in KIRC, differences in ICD patterns were found to correlate with tumor heterogeneity and therapeutic complexity. Therefore, this study is of clinical importance for the systematic assessment of tumor ICD patterns. At the same time, it allows the identification of ideal candidates with which to tailor the optimal immunotherapy strategy.

## Data availability statement

The datasets presented in this study can be found in online repositories. The names of the repository/repositories and accession number(s) can be found in the article/**Supplementary Material**.

## Author contributions

All authors contributed to the conceptualization and design of the study. LW, YRZ and ZR made substantial contributions to the acquisition of data, analysis, and interpretation of data and drafted the manuscript. WS, ZW, TZ, HL and YZ revised the manuscript critically for important intellectual content. DG, LZ and ZH have given important help to this article. All authors contributed to the article and approved the submitted version. WL, QW and GW are responsible for statistic and quality review.

## Supplementary material

Supplementary Figure 1 ICD cluster clustering analysis grouping **(A–F)** The consensus matrix of all KICD samples for each k (k=2-5), showing the cluster stability for 1000 iterations of hierarchical clustering, for the ICD cluster **(A–D)**. The cluster stability of 1000 iterations of hierarchical clustering, and its Delta area **(E)**, the consensus CDF **(F)**.

Supplementary Figure 2 Gene cluster clustering analysis grouping **(A–F)** The consensus matrix of all KICD samples for each k (k=2-5), showing the cluster stability for 1000 iterations of hierarchical clustering, for the gene cluster **(A–D)**. The cluster stability of 1000 iterations of hierarchical clustering, and its Delta area **(E)**, the consensus CDF **(F)**.

Supplementary Figure 3 Mutations in ICD-related genes **(A)** Mutation status of the TOP 30 ICD-related genes. **(B)** Statistics of specific mutation patterns of ICD-related genes. **(C)** Co-expression of high mutation-expressing ICD-related genes.
