# Supplementary figures and images for: An immunogenic cell death-related classification predicts prognosis and response to immunotherapy in kidney renal clear cell carcinoma
Source: Front Oncol. 2023 Aug 23;13:1147805. doi: 10.3389/fonc.2023.1147805 (PMC10482408; doi:10.3389/fonc.2023.1147805)

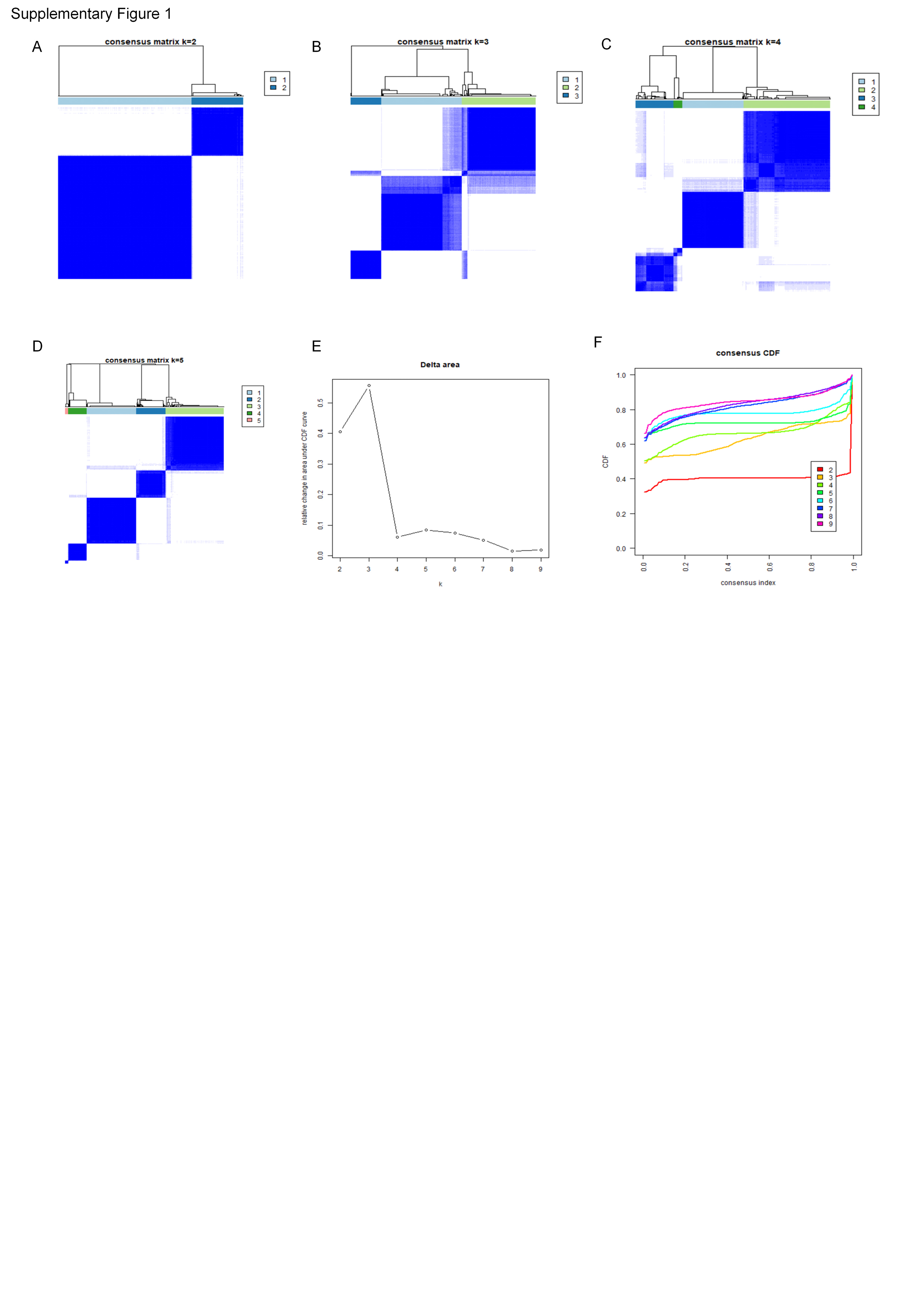

Supplement: Supplementary Figure 1 — ICD cluster clustering analysis grouping (A–F) The consensus matrix of all KICD samples for each k (k=2-5), showing the cluster stability for 1000 iterations of hierarchical clustering, for the ICD cluster (A–D). The cluster stability of 1000 iterations of hierarchical clustering, and its Delta area (E), the consensus CDF (F). [file Image_1.jpeg]

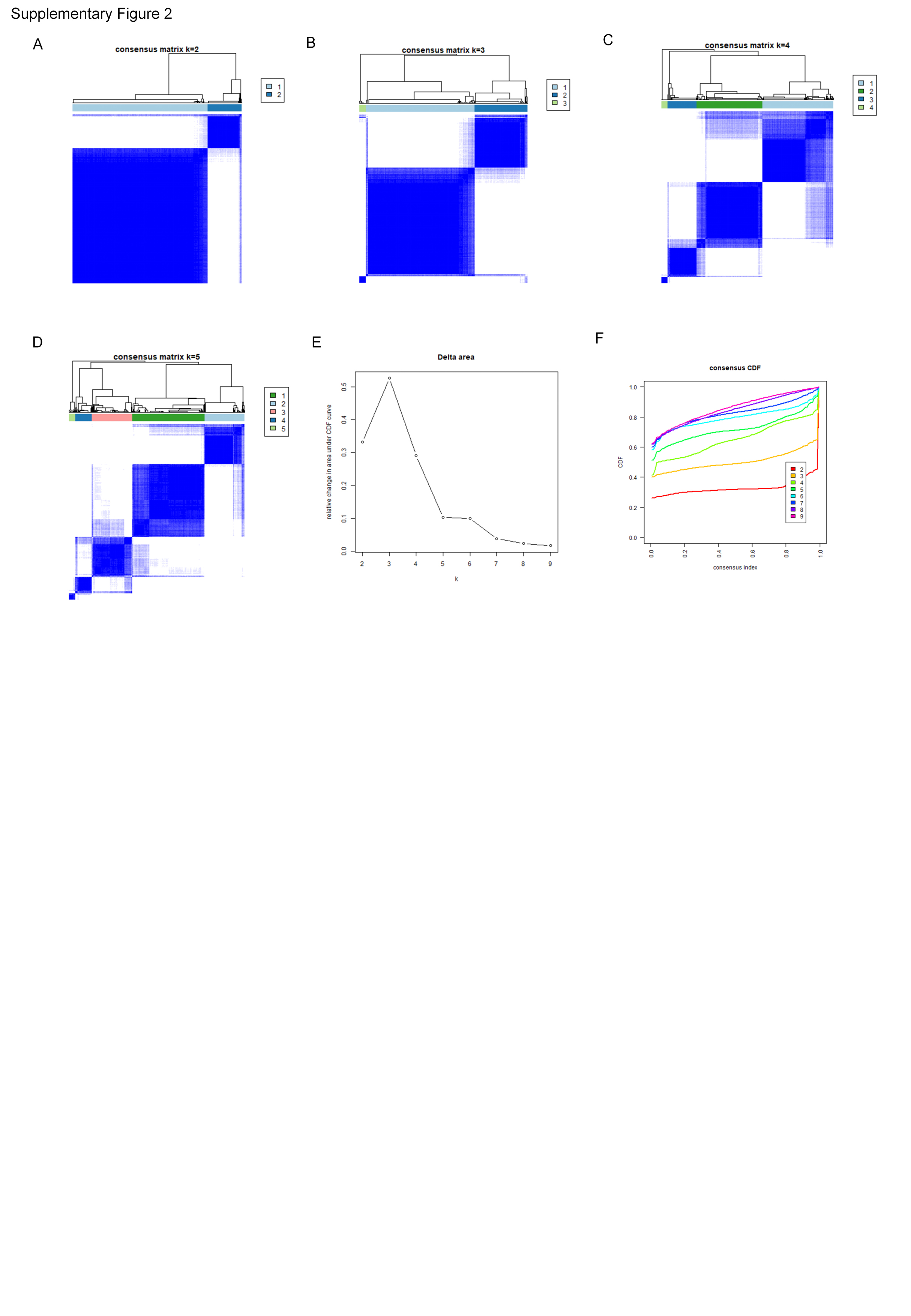

Supplement: Supplementary Figure 2 — Gene cluster clustering analysis grouping (A–F) The consensus matrix of all KICD samples for each k (k=2-5), showing the cluster stability for 1000 iterations of hierarchical clustering, for the gene cluster (A–D). The cluster stability of 1000 iterations of hierarchical clustering, and its Delta area (E), the consensus CDF (F). [file Image_2.jpeg]

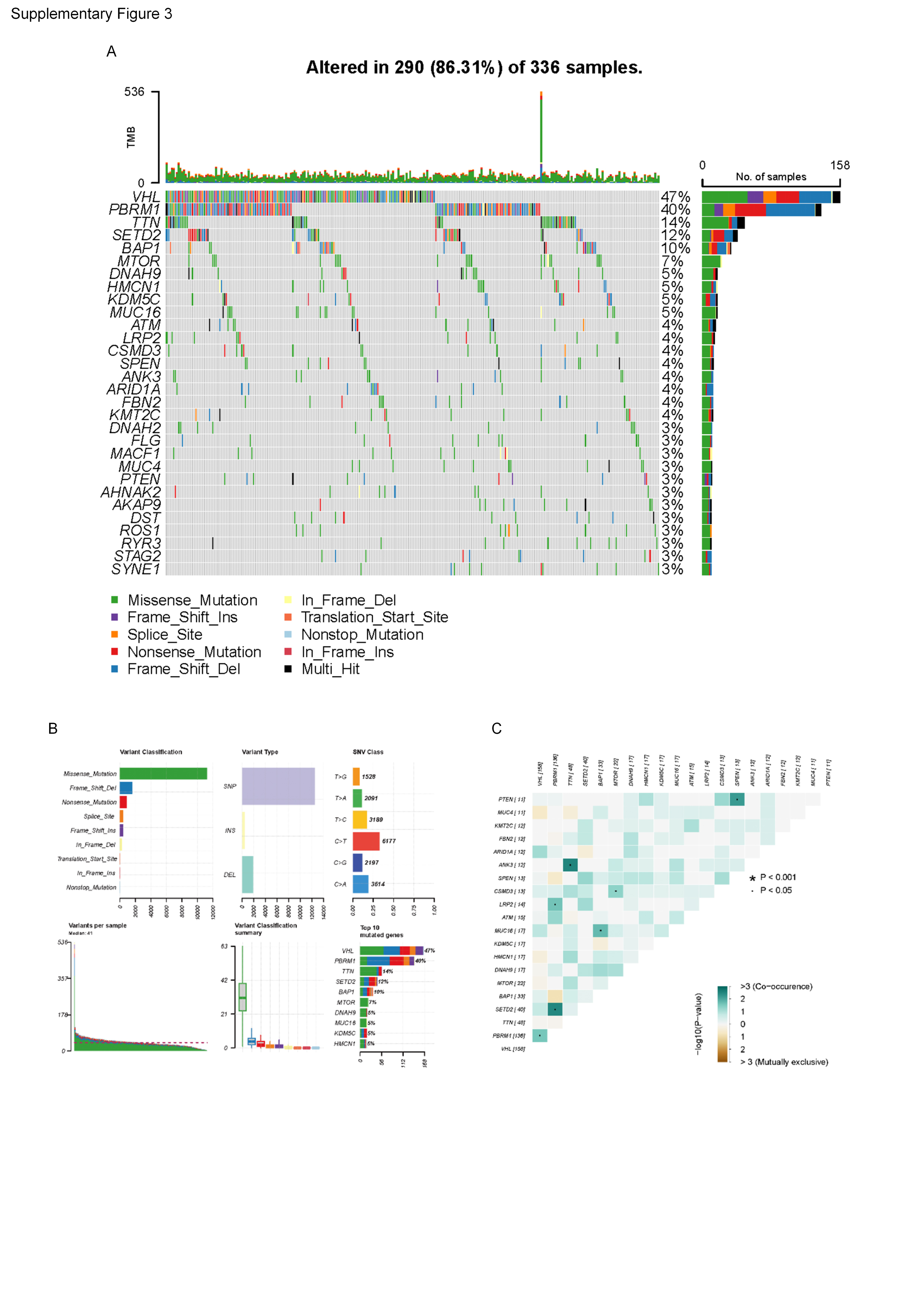

Supplement: Supplementary Figure 3 — Mutations in ICD-related genes (A) Mutation status of the TOP 30 ICD-related genes. (B) Statistics of specific mutation patterns of ICD-related genes. (C) Co-expression of high mutation-expressing ICD-related genes. [file Image_3.jpeg]
