# Supplementary material for: An immunogenic cell death-related classification predicts prognosis and response to immunotherapy in kidney renal clear cell carcinoma
Source: Front Oncol. 2023 Aug 23;13:1147805. doi: 10.3389/fonc.2023.1147805 (PMC10482408; doi:10.3389/fonc.2023.1147805)
Supplement: Supplementary file 4 [file DataSheet_1.docx]

https://www.jianguoyun.com/p/DTb6TWQQ3KKqCxi6jfQEIAA
